# Supplementary material for: Association of Upper Lip Morphology Characteristics with Sagittal and Vertical Skeletal Patterns: A Cross Sectional Study
Source: Diagnostics (Basel). 2021 Sep 18;11(9):1713. doi: 10.3390/diagnostics11091713 (PMC8471513; doi:10.3390/diagnostics11091713)
Supplement: Supplementary file 1 [file diagnostics-11-01713-s001.zip › diagnostics-1359430-supplementary/Supplementary Materials/Table s1.pdf]

**Table S1.** Demographic and clinical characteristics of samples involved in this study stratified by vertical skeletal pattern.

| Level | Overall | Normodivergent | Hyperdivergent | Hypodivergent | P-value |
|---|---|---|---|---|---|
| **N** | 2079 | 1314 | 239 | 526 | |
| **Age (%)** | | | | | |
| Adolescent | 734(35.3) | 498(37.9) | 70(29.3) | 166(31.6) | 0.008 |
| Young adult | 1272(61.2) | 765(58.2) | 162(67.8) | 345(65.6) | |
| Middle age | 73(3.5) | 51(3.9) | 7(2.9) | 15(2.9) | |
| **Gender (%)** | | | | | |
| Male | 623(30.0) | 378(28.8) | 31(13.0) | 214(40.7) | <0.001 |
| Female | 1456(70.0) | 936(71.2) | 208(87.0) | 312(59.3) | |
| **Molar Relationship (%)** | | | | | |
| I | 654(31.5) | 445(33.9) | 71(29.7) | 138(26.2) | <0.001 |
| II-1 | 654(31.5) | 401(30.5) | 60(25.1) | 193(36.7) | |
| II-2 | 205(9.9) | 123(9.4) | 41(17.2) | 41(7.8) | |
| III | 506(24.3) | 307(23.4) | 58(24.3) | 141(26.8) | |
| IV | 60(2.9) | 38(2.9) | 9(3.8) | 13(2.5) | |
| **Upper crowding (%)** | | | | | |
| I | 1275(61.3) | 782(59.5) | 143(59.8) | 350(66.5) | 0.061 |
| II | 507(24.4) | 335(25.5) | 57(23.8) | 115(21.9) | |
| III | 297(14.3) | 197(15.0) | 39(16.3) | 61(11.6) | |
| **Lower crowding (%)** | | | | | |
| I | 1325(63.7) | 816(62.1) | 143(59.8) | 366(69.6) | 0.013 |
| II | 557(26.8) | 366(27.9) | 67(28.0) | 124(23.6) | |
| III | 197(9.5) | 132(10.0) | 29(12.1) | 36(6.8) | |
| **Overbite (%)** | | | | | |
| Normal | 1302(62.6) | 847(64.5) | 122(51.0) | 333(63.3) | <0.001 |
| Deep | 246(11.8) | 114(8.7) | 13(5.4) | 119(22.6) | |
| Open | 117(5.6) | 66(5.0) | 44(18.4) | 7(1.3) | |

| | | | | | |
|---|---|---|---|---|---|
| Shallow | 414(19.9) | 287(21.8) | 60(25.1) | 67(12.7) | |
| **Overjet (%)** | | | | | |
| Normal | 1125(54.1) | 739(56.2) | 126(52.7) | 260(49.4) | <0.001 |
| Cross | 234(11.3) | 117(8.9) | 28(11.7) | 89(16.9) | |
| Deep | 690(33.2) | 437(33.3) | 81(33.9) | 172(32.7) | |
| Shallow | 30(1.4) | 21(1.6) | 4(1.7) | 5(1.0) | |
| **Sagittal skeletal pattern (%)** | | | | | |
| I | 968(46.6) | 636(48.4) | 84(35.1) | 248(47.1) | <0.001 |
| II | 691(33.2) | 454(34.6) | 128(53.6) | 109(20.7) | |
| III | 420(20.2) | 224(17.0) | 27(11.3) | 169(32.1) | |
| **U1-ANS (mm) (mean (SD))** | 27.60(3.08) | 27.89(2.90) | 29.45(2.67) | 26.05(3.04) | <0.001 |
| **U1-OP (mean (SD))** | 54.09(7.65) | 53.61(7.35) | 52.89(6.63) | 55.81(8.52) | <0.001 |
| **U1-PP (mm) (mean (SD))** | 27.28(3.12) | 27.56(2.94) | 29.21(2.69) | 25.69(3.06) | <0.001 |
| **U1-NA (mean (SD))** | 27.92(8.76) | 27.80(8.47) | 25.47(7.99) | 29.36(9.51) | <0.001 |
| **U1-NA (mm) (mean (SD))** | 5.65(2.85) | 5.70(2.82) | 5.31(2.67) | 5.68(2.99) | 0.152 |
| **U1-SN (mean (SD))** | 108.46(9.48) | 108.10(9.04) | 103.11(8.07) | 111.78(9.88) | <0.001 |
| **U1-PP (mean (SD))** | 119.54(8.88) | 119.40(8.59) | 116.90(7.86) | 121.07(9.71) | <0.001 |
| **U1-NPo (mm) (mean (SD))** | 9.86(4.92) | 10.31(4.62) | 12.64(5.44) | 7.48(4.39) | <0.001 |
| **UL-EP (mm) (mean (SD))** | 0.53(2.85) | 0.73(2.76) | 1.41(2.99) | -0.38(2.81) | <0.001 |
| **Upper lip to S line (mean (SD))** | 4.60(2.64) | 4.55(2.53) | 3.92(2.86) | 5.04(2.74) | <0.001 |
| **Upper Lip Length (ULL) (mm) (mean (SD))** | 21.46(2.40) | 21.55(2.31) | 22.43(2.23) | 20.78(2.51) | <0.001 |
| **Basic upper lip thickness (mm) (mean (SD))** | 14.48(1.97) | 14.40(1.95) | 14.33(1.90) | 14.74(2.02) | 0.002 |
| **Nasolabial A (mean (SD))** | 95.74(11.83) | 96.48(11.52) | 97.57(11.50) | 93.05(12.32) | <0.001 |
| **Upper lip thickness (mm) (mean (SD))** | 14.85(2.50) | 14.80(2.44) | 14.80(2.49) | 14.99(2.65) | 0.329 |
| **Superior sulcus depth (mm) (mean (SD))** | 4.84(2.17) | 4.75(2.12) | 4.34(2.17) | 5.28(2.23) | <0.001 |
