# Supplementary material for: Association of Upper Lip Morphology Characteristics with Sagittal and Vertical Skeletal Patterns: A Cross Sectional Study
Source: Diagnostics (Basel). 2021 Sep 18;11(9):1713. doi: 10.3390/diagnostics11091713 (PMC8471513; doi:10.3390/diagnostics11091713)
Supplement: Supplementary file 1 [file diagnostics-11-01713-s001.zip › diagnostics-1359430-supplementary/Supplementary Materials/Table s2.pdf]

Table S2. Demographic and clinical characteristics of samples involved in this study stratified by sagittal and vertical skeletal pattern.

Level	Overall	I-Hyper	I-Hypo	I-Norm	II-Hyper	II-Hypo	II-Norm	III-Hyper	III-Hypo	III-Norm	P
**N**	2079	84	248	636	128	109	454	27	169	224	
**Age (%)**											
Adolescent	734(35.3)	30(35.7)	86(34.7)	257(40.4)	32(25.0)	38(34.9)	178(39.2)	8(29.6)	42(24.9)	63(28.1)	<0.001
Young_adult	1272(61.2)	52(61.9)	155(62.5)	359(56.4)	92(71.9)	67(61.5)	250(55.1)	18(66.7)	123(72.8)	156(69.6)	
Middle_age	73(3.5)	2(2.4)	7(2.8)	20(3.1)	4(3.1)	4(3.7)	26(5.7)	1(3.7)	4(2.4)	5(2.2)	
**Gender (%)**											
Male	623(30.0)	18(21.4)	85(34.3)	168(26.4)	10(7.8)	50(45.9)	139(30.6)	3(11.1)	79(46.7)	71(31.7)	<0.001
Female	1456(70.0)	66(78.6)	163(65.7)	468(73.6)	118(92.2)	59(54.1)	315(69.4)	24(88.9)	90(53.3)	153(68.3)	
**Molar Relationship (%)**											
I	654(31.5)	25(29.8)	83(33.5)	268(42.1)	40(31.2)	19(17.4)	127(28.0)	6(22.2)	36(21.3)	50(22.3)	<0.001
II_1	654(31.5)	19(22.6)	105(42.3)	164(25.8)	41(32.0)	63(57.8)	214(47.1)	0(0.0)	25(14.8)	23(10.3)	
II_2	205(9.9)	7(8.3)	22(8.9)	48(7.5)	34(26.6)	14(12.8)	72(15.9)	0(0.0)	5(3.0)	3(1.3)	
III	506(24.3)	27(32.1)	30(12.1)	140(22.0)	11(8.6)	10(9.2)	31(6.8)	20(74.1)	101(59.8)	136(60.7)	
IV	60(2.9)	6(7.1)	8(3.2)	16(2.5)	2(1.6)	3(2.8)	10(2.2)	1(3.7)	2(1.2)	12(5.4)	
**Upper crowding (%)**											
I	1275(61.3)	49(58.3)	167(67.3)	374(58.8)	82(64.1)	84(77.1)	280(61.7)	12(44.4)	99(58.6)	128(57.1)	0.05
II	507(24.4)	21(25.0)	56(22.6)	168(26.4)	27(21.1)	14(12.8)	111(24.4)	9(33.3)	45(26.6)	56(25.0)	
III	297(14.3)	14(16.7)	25(10.1)	94(14.8)	19(14.8)	11(10.1)	63(13.9)	6(22.2)	25(14.8)	40(17.9)	
**Lower crowding (%)**											
I	1325(63.7)	51(60.7)	170(68.5)	387(60.8)	71(55.5)	64(58.7)	257(56.6)	21(77.8)	132(78.1)	172(76.8)	<0.001
II	557(26.8)	26(31.0)	57(23.0)	187(29.4)	37(28.9)	34(31.2)	139(30.6)	4(14.8)	33(19.5)	40(17.9)	
III	197(9.5)	7(8.3)	21(8.5)	62(9.7)	20(15.6)	11(10.1)	58(12.8)	2(7.4)	4(2.4)	12(5.4)	
**Overbite (%)**											
Normal	1302(62.6)	43(51.2)	173(69.8)	431(67.8)	72(56.2)	74(67.9)	317(69.8)	7(25.9)	86(50.9)	99(44.2)	<0.001
Deep	246(11.8)	3(3.6)	51(20.6)	37(5.8)	9(7.0)	32(29.4)	71(15.6)	1(3.7)	36(21.3)	6(2.7)	
Open	117(5.6)	14(16.7)	1(0.4)	25(3.9)	22(17.2)	1(0.9)	16(3.5)	8(29.6)	5(3.0)	25(11.2)	
Shallow	414(19.9)	24(28.6)	23(9.3)	143(22.5)	25(19.5)	2(1.8)	50(11.0)	11(40.7)	42(24.9)	94(42.0)	
**Overjet (%)**											

Normal	1125(54.1)	61(72.6)	148(59.7)	429(67.5)	59(46.1)	43(39.4)	197(43.4)	6(22.2)	69(40.8)	113(50.4)	<0.001
Cross	234(11.3)	8(9.5)	4(1.6)	24(3.8)	1(0.8)	0(0.0)	4(0.9)	19(70.4)	85(50.3)	89(39.7)	
Deep	690(33.2)	14(16.7)	95(38.3)	175(27.5)	67(52.3)	66(60.6)	252(55.5)	0(0.0)	11(6.5)	10(4.5)	
Shallow	30(1.4)	1(1.2)	1(0.4)	8(1.3)	1(0.8)	0(0.0)	1(0.2)	2(7.4)	4(2.4)	12(5.4)	
**U1-ANS (mm) (mean (SD))**	27.60(3.08)	29.61(2.68)	26.40(2.83)	27.70(2.82)	29.55(2.58)	26.82(2.89)	28.60(2.84)	28.49(2.95)	25.04(3.18)	26.97(2.92)	<0.001
**U1-OP (mean (SD))**	54.09(7.65)	53.36(4.96)	56.27(8.16)	53.48(7.24)	52.88(7.77)	55.99(9.66)	54.34(8.00)	51.47(5.03)	55.03(8.25)	52.53(6.03)	<0.001
**U1-PP (mm) (mean (SD))**	27.28(3.12)	29.40(2.64)	26.05(2.87)	27.38(2.86)	29.27(2.64)	26.43(2.93)	28.29(2.88)	28.29(2.95)	24.69(3.17)	26.60(2.93)	<0.001
**U1-NA (mean (SD))**	27.92(8.76)	26.42(6.44)	27.94(9.24)	28.33(7.84)	23.91(8.90)	25.95(9.80)	24.95(8.94)	29.92(5.40)	33.64(8.17)	32.04(7.04)	<0.001
**U1-NA (mm) (mean (SD))**	5.65(2.85)	5.68(2.82)	5.39(2.96)	5.85(2.66)	4.77(2.51)	4.80(2.58)	4.78(2.66)	6.75(2.20)	6.67(3.03)	7.12(2.93)	<0.001
**U1-SN (mean (SD))**	108.46(9.48)	102.93(6.58)	110.64(9.47)	108.25(8.76)	102.49(9.07)	110.26(11.13)	106.48(9.77)	106.56(6.44)	114.44(9.10)	110.96(7.44)	<0.001
**U1-PP (mean (SD))**	119.54(8.88)	116.92(6.62)	119.78(9.31)	119.52(8.41)	116.31(8.86)	119.74(10.90)	117.96(9.14)	119.61(5.77)	123.82(8.91)	121.99(7.20)	<0.001
**U1-NPo (mm) (mean (SD))**	9.86(4.92)	10.75(3.88)	8.04(3.85)	9.78(3.48)	15.64(4.25)	11.30(3.81)	13.58(3.93)	4.33(2.69)	4.19(2.87)	5.21(3.37)	<0.001
**UL-EP (mm) (mean (SD))**	0.53(2.85)	0.85(2.56)	0.17(2.42)	0.50(2.24)	2.67(2.46)	1.66(2.08)	2.42(2.31)	-2.82(1.96)	-2.51(2.35)	-2.03(2.44)	<0.001
**Upper lip to S line (mean (SD))**	4.60(2.64)	4.20(2.91)	4.84(2.74)	4.64(2.45)	3.83(2.67)	4.77(2.40)	4.23(2.49)	3.47(3.54)	5.52(2.88)	4.93(2.76)	<0.001
**Upper Lip Length (ULL) (mm) (mean (SD))**	21.46(2.40)	22.55(1.97)	20.91(2.15)	21.45(2.25)	22.57(2.39)	21.90(2.35)	22.11(2.25)	21.39(1.98)	19.86(2.78)	20.71(2.29)	<0.001
**Basic upper lip thickness (mm) (mean (SD))**	14.48(1.97)	14.76(1.72)	14.44(1.84)	14.37(1.82)	13.84(1.85)	14.37(2.09)	14.17(2.01)	15.26(2.04)	15.43(2.07)	14.98(2.06)	<0.001
**Nasolabial A (mean**	95.74(11.83)	95.62(10.56)	94.46(12.10)	96.34(11.10)	99.26(11.28)	97.95(9.99)	99.43(11.19)	95.67(14.26)	87.81(12.18)	90.90(11.28)	<0.001

**(SD))**										
**Upper lip thickness (mm) (mean (SD))**	14.85(2.50)	15.60(2.49)	14.67(2.54)	14.75(2.36)	14.08(2.21)	14.22(2.19)	14.54(2.44)	15.73(2.73)	15.96(2.81)	15.48(2.55)	<0.001
**Superior sulcus depth (mm) (mean (SD))**	4.84(2.17)	4.64(2.12)	5.09(2.24)	4.79(2.14)	4.11(2.21)	4.86(2.20)	4.44(2.09)	4.48(2.05)	5.84(2.15)	5.27(2.03)	<0.001
