# Supplementary material for: Association of Upper Lip Morphology Characteristics with Sagittal and Vertical Skeletal Patterns: A Cross Sectional Study
Source: Diagnostics (Basel). 2021 Sep 18;11(9):1713. doi: 10.3390/diagnostics11091713 (PMC8471513; doi:10.3390/diagnostics11091713)
Supplement: Supplementary file 1 [file diagnostics-11-01713-s001.zip › diagnostics-1359430-supplementary/Supplementary Materials/Table s3.pdf]

Table S3. Adjusted values of upper lip characteristics in sagittal and vertical skeletal patterns.

Level	Overall	Class I	Class II	Class III	P-value
**N**	2079	968	691	420	
**Nasolabial A (mean (SD))**	95.74 (8.74)	95.80 (7.93)	99.17 (7.91)	89.96 (8.80)	<0.001
**Upper Lip Length (ULL) (mm) (mean (SD))**	21.46 (1.93)	21.41 (1.77)	22.16 (1.85)	20.41 (1.90)	<0.001
**Basic upper lip thickness (mm) (mean (SD))**	14.48 (1.59)	14.40 (1.58)	14.33 (1.50)	14.74 (1.63)	<0.001
**Upper lip thickness (mm) (mean (SD))**	14.85 (2.07)	14.80 (2.04)	14.40 (1.87)	15.69 (2.20)	<0.001
**Superior sulcus depth (mm) (mean (SD))**	4.84 (2.02)	4.85 (2.01)	4.44 (1.92)	5.45 (2.05)	<0.001
**Level**	**Overall**	**Normodivergent**	**Hyperdivergent**	**Hypodivergent**	**P-value**
**N**	2079	1314	239	526	
**Nasolabial A (mean (SD))**	95.74 (8.74)	96.48 (8.37)	97.57 (8.45)	93.05 (9.19)	<0.001
**Upper Lip Length (ULL) (mm) (mean (SD))**	21.46 (1.93)	21.55 (1.83)	22.43 (1.88)	20.78 (1.95)	<0.001
**Basic upper lip thickness (mm) (mean (SD))**	14.48 (1.59)	14.42 (1.48)	14.14 (1.62)	15.18 (1.57)	<0.001
**Upper lip thickness (mm) (mean (SD))**	14.85 (2.07)	14.80 (2.00)	14.80 (2.01)	14.99 (2.27)	0.198
**Superior sulcus depth (mm) (mean (SD))**	4.84 (2.02)	4.75 (1.96)	4.34 (2.03)	5.28 (2.10)	<0.001
