# Supplementary material for: Association of Upper Lip Morphology Characteristics with Sagittal and Vertical Skeletal Patterns: A Cross Sectional Study
Source: Diagnostics (Basel). 2021 Sep 18;11(9):1713. doi: 10.3390/diagnostics11091713 (PMC8471513; doi:10.3390/diagnostics11091713)
Supplement: Supplementary file 1 [file diagnostics-11-01713-s001.zip › diagnostics-1359430-supplementary/Supplementary Materials/Table s4.pdf]

Table S4. Adjusted values of upper lip characteristics stratified by sagittal and vertical skeletal patterns.

Level	Overall	I-Hyper	I-Hypo	I-Norm	II-Hyper	II-Hypo	II-Norm	III-Hyper	III-Hypo	III-Norm	P
N	2079	84	248	636	128	109	454	27	169	224	
Nasolabial A (mean (SD))	95.74 (8.75)	95.62 (8.19)	94.46 (8.04)	96.34 (7.84)	99.26 (8.15)	97.95 (7.92)	99.43 (7.78)	95.67 (8.81)	87.81 (9.14)	90.90 (8.17)	<0.001
Upper Lip Length (ULL) (mm) (mean (SD))	21.46 (1.93)	22.55 (1.70)	20.91 (1.76)	21.45 (1.73)	22.57 (1.89)	21.90 (1.83)	22.11 (1.81)	21.39 (2.00)	19.86 (1.97)	20.71 (1.73)	<0.001
Basic upper lip thickness (mm) (mean (SD))	14.48 (1.59)	14.76 (1.33)	14.44 (1.49)	14.37 (1.49)	13.84 (1.47)	14.37 (1.71)	14.17 (1.63)	15.26 (1.32)	15.43 (1.63)	14.98 (1.55)	<0.001
Upper lip thickness (mm) (mean (SD))	14.85 (2.07)	15.60 (2.01)	14.67 (2.24)	14.75 (1.94)	14.08 (1.66)	14.22 (1.83)	14.54 (1.92)	15.73 (2.39)	15.96 (2.26)	15.48 (2.11)	<0.001
Superior sulcus depth (mm) (mean (SD))	4.84 (2.02)	4.64 (2.08)	5.09 (2.12)	4.79 (1.96)	4.11 (1.99)	4.86 (1.85)	4.44 (1.90)	4.48 (2.06)	5.84 (2.14)	5.27 (1.94)	<0.001
